# TFEB agonist clomiphene citrate activates the autophagy-lysosomal pathway and ameliorates Alzheimer's disease symptoms in mice

**DOI:** 10.1016/j.jbc.2024.107929

**Published:** 2024-10-24

**Authors:** Jieru Lin, Yi Yuan, Chunhuan Huang, Jiayu Zi, Lu Li, Jiamiao Liu, Xiaoting Wu, Wei Li, Qing Zhao, Yuyin Li, Zhenxing Liu, Aipo Diao

**Affiliations:** 1 School of Biotechnology, Tianjin University of Science and Technology, Tianjin, China; 2 School of Basic Medical Science, Inner Mongolia Medical University, Hohhot, Inner Mongolia, China

**Keywords:** TFEB, clomiphene citrate, autophagy, lysosome, Alzheimer's disease

## Abstract

Autophagy is a conserved eukaryotic cellular clearance and recycling process through the lysosome-mediated degradation of damaged organelles and protein aggregates to maintain homeostasis. Impairment of the autophagy-lysosomal pathway is implicated in the pathogenesis of Alzheimer’s disease (AD). Transcription factor EB (TFEB) is a master regulator of autophagy and lysosomal biogenesis. Therefore, activating TFEB and autophagy provides a novel strategy for AD treatment. We previously described that clomiphene citrate (CC) promotes nuclear translocation of TFEB and increases autophagy and lysosomal biogenesis. In this study, 7- and 3-month-old APP/PS1 mice were treated with TFEB agonist CC and assessed. The behavioral tests were performed using Morris water maze and open field test. Additional changes in amyloid-β pathology, autophagy, and inflammatory response were determined. We found that CC activated TFEB and the autophagy-lysosomal pathway in neuronal cells. Moreover, using mouse model of Alzheimer's disease, CC treatment promoted clearance of amyloid-β plaques and ameliorated cognitive function in both 7- and 3-month-old APP/PS1 mice. The CC-induced activation of TFEB occurs by promoting acetylation of TFEB for nuclear translocation. These findings provide a molecular mechanism for the TFEB-mediated activation of the autophagy-lysosome pathway by CC, which has the potential to be repurposed and applied in the treatment or prevention of AD.

The autophagy-lysosomal pathway (ALP), a lysosome-mediated process responsible for intracellular component degradation, plays a critical role in clearing aggregated proteins and damaged organelles (1). Mounting evidence suggests that impairment of ALP is implicated in the pathogenesis of neurodegenerative diseases including Alzheimer’s disease (AD). In AD brains, defective ALP degradation contributes to the accumulation of amyloid-β (Aβ) and hyperphosphorylated tau (p-tau), which are clinical hallmarks of AD (2, 3). Conversely, genetic or pharmacological activation of ALP effectively promotes the clearance of toxic Aβ and tau aggregates (4, 5). Furthermore, defective mitophagy in the brain tissues of human AD or transgenic AD mice leads to the accumulation of damaged mitochondria, which further exacerbates AD pathology (6). Restoration of mitophagy alleviates memory loss in AD models by inhibiting Aβ plaques and hyperphosphorylated tau (7). Thus, targeting the activation of ALP-mediated clearance of aggregated proteins and damaged organelles may be a promising approach for AD therapy.

As a master regulator of the ALP, transcription factor EB (TFEB) controls lysosomal biogenesis and autophagic induction through its binding to the CLEAR (coordinated lysosomal expression and regulation) motif, and facilitating the expression of multiple genes involved in lysosomal and autophagic processes (8, 9). Activation of TFEB occurs through the regulation of upstream kinase or phosphatase activity, such as mechanistic target of rapamycin kinase complex 1 (mTORC1), AMP-activated protein kinase (AMPK) and calcineurin. During nutrient deprivation, AMP-activated protein kinase activation and mTORC1 inhibition leads to phosphorylation of TFEB and stay in the cytoplasm (10, 11). Under the condition of lysosomal stress, calcium ion release from lysosomes activates calcineurin, facilitating its binding to TFEB and subsequent dephosphorylation, thereby promoting TFEB nuclear translocation and consequently enhancing the expression of genes involved in autophagy and lysosomal function (12). Besides phosphorylation and dephosphorylation, several other protein modifications also regulate nuclear translocation and activation of TFEB, including acetylation and ubiquitination (13).

Overexpression of TFEB can alleviate AD progression by reducing Aβ accumulation and Aβ-induced reactive oxygen species production (14). TFEB enhances lysosomal biogenesis in both astrocytes and neurons, leading to increased Aβ clearance in astrocytes and reduced Aβ generation in neurons (15). Compound C1, an analog of curcumin, could induce ALP activation by directly stimulating TFEB, thereby alleviating Aβ precursor protein (APP) and Tau pathology during the early stages of AD progression *in vivo* (16). Furthermore, trehalose facilitates the nuclear translocation of TFEB independent of mTORC1 activity, and its administration may reduce disease burden in a mouse model of a prototypical neurodegenerative disease (17). Therefore, modulating TFEB activation with small-molecule activators to enhance cytoprotective ALP activity is a potential treatment for AD.

Our previous study indicated that clomiphene citrate (CC), a Food and Drug Administration (FDA) approved selective estrogen receptor modulator, promotes nuclear translocation of TFEB and increases autophagy and lysosomal biogenesis in an estrogen receptor independent manner (18). Despite these observations, the specific role of CC in AD treatment remains to be fully elucidated. Considering the role of TFEB in modulating AD-related phenotypes, in this study, we investigated the molecular mechanisms by which CC activates TFEB and explored its potential therapeutic role in AD, and found that CC treatment promoted clearance of Aβ plaques and improved cognitive function in the APP/PS1 mouse. Our findings provide insights into understanding the function of TFEB agonist CC and its potential use as a pharmacological therapy for AD treatment.

## Results

### Clomiphene citrate activates TFEB and the autophagy-lysosome pathway in neuronal cells

**Figure 1:**
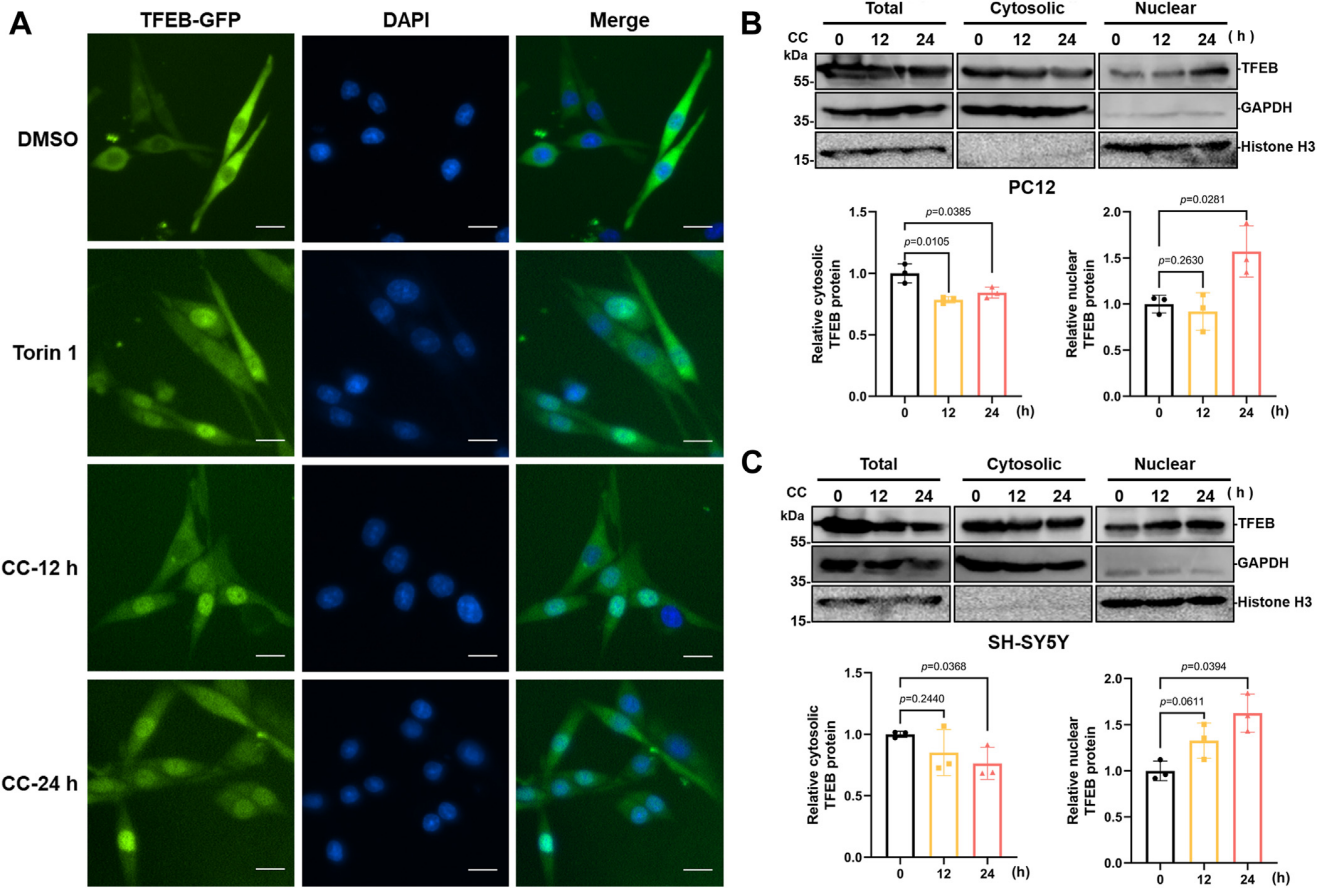
**CC induces TFEB nuclear translocation in PC12 and SH-SY5Y cells**. *A*, analysis of TFEB-GFP nuclear translocation. PC12 cells stably expressing a TFEB-GFP were cultured in medium with or without 10 μM CC for indicated time points, and observed using fluorescence microscopy. Nuclei were stained using DAPI (*blue*). Torin1 (200 nM, 1 h) was used as a positive control. The scale bar represents 20 μm. To detect the effect of CC on intracellular localization of endogenous TFEB, PC12 (*B*), or SH-SY5Y (*C*) cells were treated with or without 10 μM CC for indicated time points. Western blot was used to detect endogenous TFEB protein levels in the nuclear and cytosolic fractions. GAPDH and histone H3 were used as the loading controls. Lower panels indicate densitometric analysis of Western blot using ImageJ software. The protein levels of TFEB were normalized to levels of the cytosolic marker GAPDH or nuclear marker histone H3. Data are presented as mean ± SD of three independent experiments. CC, clomiphene citrate; DAPI, 4′,6-diamidino-2-phenylindole; TFEB, transcription factor EB.

We previously reported that CC promotes nuclear translocation of TFEB and increases autophagy and lysosomal biogenesis in HeLa and MDA-MB-231 cancer cells. Duo to both HeLa and MDA-MB-231 are estrogen receptor-negative cell lines, CC-induced activation of TFEB and autophagy is estrogen receptor independent (18). In order to investigate whether CC also activates TFEB in neuronal cells, firstly, rat adrenal pheochromocytoma cell line PC12 cells stably expressing a TFEB-GFP fusion protein were generated. Significant nuclear translocation of TFEB-GFP was observed in PC12 cells after treatment with 10 μM CC for 12 or 24 h, compared to the control (Fig. 1*A*), and similar results were also observed using the mTORC1 inhibitor Torin1. The cytotoxicity of CC against PC12 cells and human neuroblastoma cell line SH-SY5Y cells was further assessed using an 3-(4,5-dimethylthiazol-2-yl)-2,5-diphenyltetrazolium bromide (MTT) assay and indicated treatment with less than 10 μM CC for 24 h did not show cytotoxicity (Fig. S1*A*). Moreover, the nuclear translocation of endogenous TFEB in response to CC treatment was evaluated by cytosolic and nuclear protein fractionation assay. The protein levels of nuclear TFEB in PC12 and SH-SY5Y cells were significantly increased after 10 μM CC treatment (Fig. 1, *B* and *C*).

**Figure 2:**
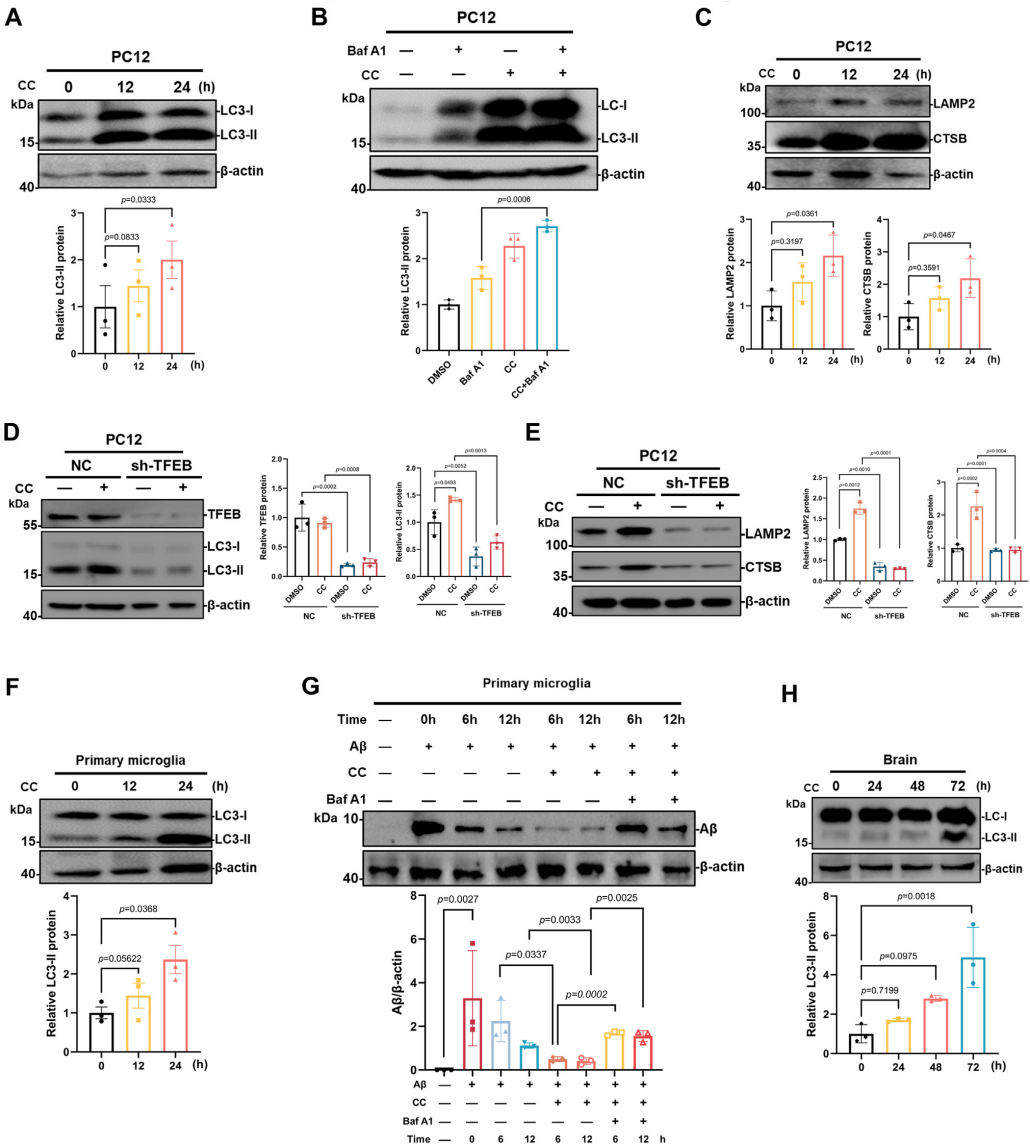
**CC activates the autophagy-lysosome pathway**. *A*, effects of CC on the protein levels of LC3-II. PC12 cells were treated with 10 μM CC for 12 h or 24 h. The levels of LC3 were measured by Western blot using antibodies against LC3. LC3-II protein level was quantified using ImageJ analysis and represented as the mean band intensity normalized to β-actin. *B*, following 10 μM CC treatment for 12 h, the cells were treated with or without 200 nM Baf A1 for an additional 2 h. LC3 protein levels were detected by Western blot. LC3-II protein level was quantified using ImageJ analysis and represented as the mean band intensity normalized to β-actin. *C*, expression of LAMP2, CTSB in 10 μM CC treated PC12 cells were measured by Western blot. Quantitative analysis of the immunoblotted proteins was performed using ImageJ and represented as the mean band intensity normalized to β-actin. *D*, PC12 cells stably expressing TFEB shRNA (sh-TFEB) or negative control shRNA (NC) were treated with or without 10 μM CC for 24 h. The levels of TFEB and LC3-II were measured by Western blot using antibodies against TFEB and LC3-II. β-actin was used as a loading control. *E*, the protein levels of LAMP2 and CTSB were detected by Western blot in the NC or sh-TFEB expressing PC12 cells. ImageJ was performed for quantitative analysis of Western blot, and β-actin was used as loading control. *F*, primary microglia cells isolated from mouse brain were treated with 10 μM CC for 12 or 24 h and analyzed by Western blot using antibodies against LC3. LC3-II protein level was quantified using ImageJ analysis and represented as the mean band intensity normalized to β-actin. *G*, primary microglia cells were pretreated with 0.3 μg/μl Aβ_42_ peptides for 24 h to allow endocytosis of Aβ_42_, after washing, cells were further treated with or without 10 μM CC for 6, 12 h, then analyzed by Western blot using antibodies against Aβ. Aβ protein level was quantified using ImageJ analysis and represented as the mean band intensity normalized to β-actin. *H*, mice were administered intraperitoneally with CC (7 mg/kg), 24, 48 and 72 h after treatment, brains were extracted and used for Western blot analysis. LC3-II expression was quantified using ImageJ analysis and represented as the mean band intensity normalized to β-actin. Data are presented as mean ± SD of three independent experiments. Aβ, amyloid-β; Baf A1, bafilomycin A1; CC, clomiphene citrate; CTSB, cathepsin B; LC3, microtubule-associated protein light chain-3; TFEB, transcription factor EB.

The cytosolic microtubule-associated protein 1A/1B-light chain 3 (LC3) is conjugated to phosphatidylethanolamine to form lipidated LC3 (LC3-II) upon the induction of autophagy, which subsequently binds to the autophagosome membrane. Therefore, the protein level of LC3-II is widely used as a marker for assessing autophagy activity. We further analyzed the role of CC in regulating autophagy-lysosome activation in neuronal cells. PC12 or SH-SY5Y cells were treated with 10 μM CC for 12 or 24 h, Western blot analysis showed that CC treatment resulted in a significant increase in the protein level of LC3-II in a time-dependent manner (Figs. 2*A* and S1*B*). Autophagy flux involves autophagosome formation, cargo sequestration, and fusion with lysosomes for degradation, bafilomycin A1 (Baf A1) inhibits autophagic degradation by blocking lysosomal acidification and is commonly used to assess autophagic flux. Furthermore, cotreatment with the autophagic degradation inhibitor Baf A1 and CC resulted in accumulation of LC3-II compared to Baf A1 alone, suggesting CC increased autophagic flux (Figs. 2*B* and S1*C*). In addition, Western blot analysis showed increased protein levels of lysosomal protein LAMP2 and CTSB (cathepsin B) in the CC-treated PC12 and SH-SY5Y cells (Figs. 2*C* and S1*D*). Moreover, shRNA-mediated knockdown of TFEB significantly decreased the CC-induced accumulation of LC3-II in PC12 cells (Fig. 2*D*), as well as the LAMP2 and CTSB protein levels in the CC-treated cells (Fig. 2*E*), suggesting TFEB is involved in CC-induced autophagy and lysosome biogenesis in PC12 cells. To further verify the effect of CC on the transcriptional activity of TFEB, quantitative reverse transcription PCR (qRT-PCR) was performed and demonstrated that the mRNA levels of TFEB downstream genes encoding autophagy related proteins (LC3B, p62) and lysosomal proteins (LAMP2, CTSB, and CTSD) in PC12 and SH-SY5Y were significantly increased following CC treatment (Fig. S1*E*). These results suggest that CC promotes autophagy and lysosomal biogenesis by activating TFEB.

Next, primary microglia cells isolated from mice brain were treated with 10 μM CC for 12 or 24 h and analyzed by Western blot, which showed the protein level of LC3-II was significantly increased after CC treatment (Fig. 2*F*), suggesting enhanced autophagy. Microglia are the major phagocytic cells in the brain and have a central role in the clearance of Aβ, however, the efficacy of this removal diminishes in AD (19). To address the effect of CC on protein degradation, primary microglia cells were pretreated with Aβ_42_ peptide to allow endocytosis of Aβ_42_, then further treated with CC, which showed that CC treatment accelerates the degradation of Aβ_42_ in microglia (Fig. 2*G*). However, adding Baf A1 attenuated CC-induced degradation of Aβ_42_, which suggests that CC promotes Aβ_42_ degradation in microglia *via* activating the ALP. Furthermore, mice were intraperitoneally administered with CC (7 mg/kg), and after treatment of 24, 48, and 72 h, mice brains were isolated and assessed for autophagy. Western blot analysis indicated that the protein level of LC3-II was increased after CC treatment (Fig. 2*H*), suggesting CC promotes autophagy in the brain of mice.

### Clomiphene citrate treatment of 7-month-old APP/PS1 mice promotes clearance of Aβ plaques and improves AD symptoms

**Figure 3:**
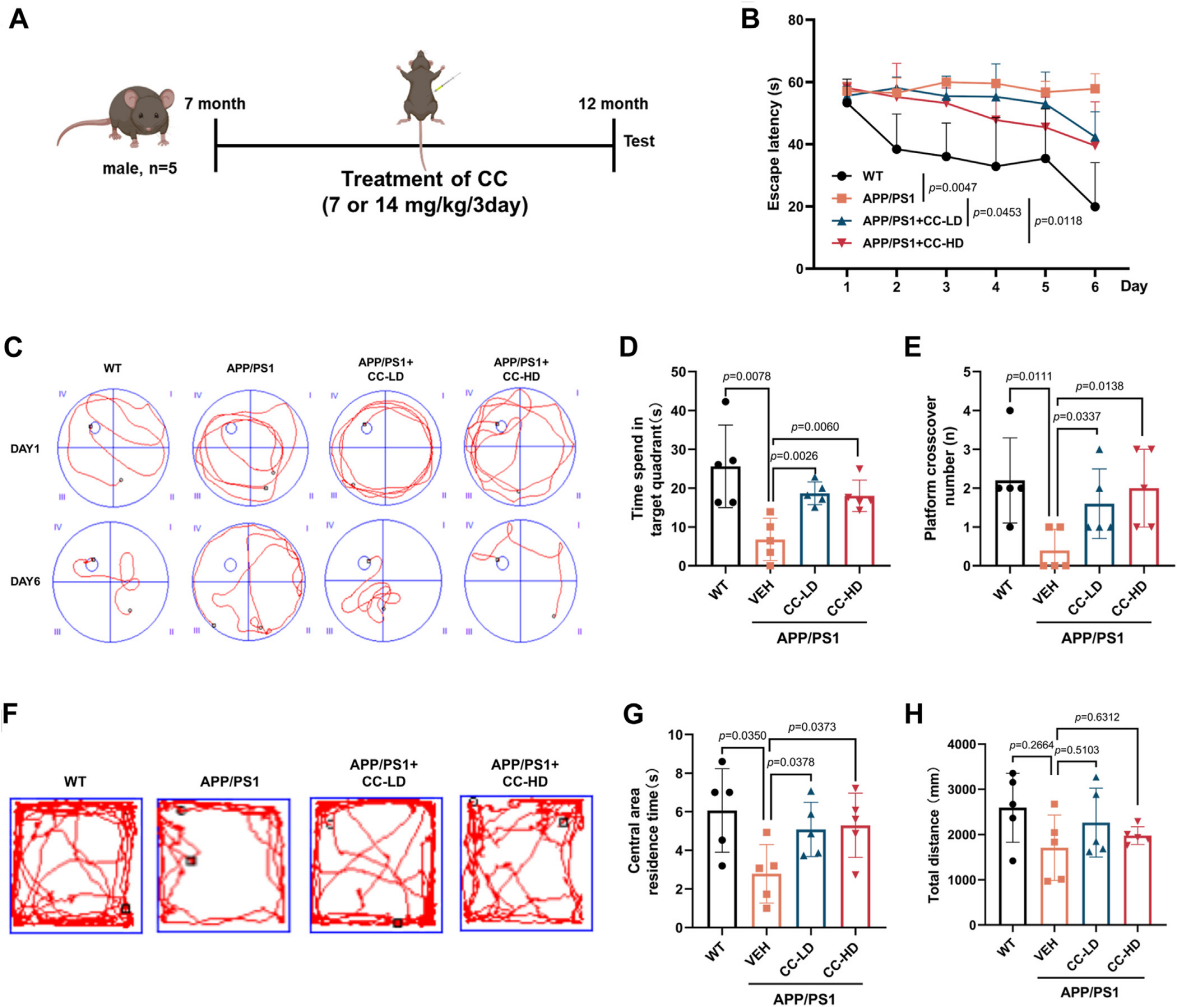
**CC treatment of 7-month-old APP/PS1 AD mice improves cognitive impairment**. The Morris water maze (MWM) and open field test were used for behavioral tests. *A*, schedule of animal treatments and experimental protocols. Briefly, 7-month-old male APP/PS1 mice were administered intraperitoneally with CC (7, 14 mg/kg body weight) or vehicle (0.9% normal saline) every 3 days to the 12 months of age before testing. CC-LD: 7 mg/kg, CC-HD: 14 mg/kg. The C57BL/6 mice were used as the WT control. n = 5. *B*, latency to escape to a hidden platform during a 6-days MWM training trial period. *C*, representative moving patterns of mice in each group during the MWM training trial (Day 1 and Day 6). Further MWM probe trial analysis, time spent in the target quadrant (*D*), and number of times that mice passed through the platform location (*E*). *F*, representative paths of mice in each group using the open field test. Quantification of the open field test for time stayed in the center area (*G*), and total distance traveled in the open field (*H*). Data are presented as mean ± SD of three independent experiments. AD, Alzheimer’s disease; APP, Aβ precursor protein; CC, clomiphene citrate; MWM, Morris water maze.

For the APP/PS1 mice, Aβ deposition begins at 3 to 4 months of age in the hippocampus (20), with cognitive deficits in spatial learning and memory in the Morris water maze (MWM) reported at 7 months (21). We also tested the Aβ levels in the brain of APP/PS1 mice at different ages using fluorescence microscopy and Western blot analysis (Fig. S2). Consistently, though Aβ could be detected by 3 months of age, clear Aβ plaques were observed in the brain at 6 months, and larger Aβ plaques were formed at 9 and 12 months. To investigate the potential therapeutic role of the TFEB agonist CC in Alzheimer's disease, firstly, 7-month-old APP/PS1 mice were treated with CC (7, 14 mg/kg) every 3 days to the 12 months of age (Fig. 3*A*). Progressive cognitive decline and increased anxiety are two important clinical hallmarks observed in AD patients (22), and therefore behavioral tests were first performed. During training trials using the classical MWM test, CC treated AD mice took less time to locate the hidden platform (Fig. 3*B*) and traveled a shorter distance to find the hidden platform (Fig. 3*C*) compared to the vehicle control. During the probe trial in MWM test, CC-treated AD mice spent more time in the target quadrant (Fig. 3*D*) and crossed the platform significantly more often (Fig. 3*E*). The MWM test indicates that CC treatment significantly improves cognition of the AD mice. It has been reported that increased anxiety is also associated with AD symptoms (23). Further open field test showed that compared to the WT mice, AD mice spent significantly less time in the open area and more time closer to the walls, indicating anxious behavior. After CC treatment, AD mice spent significantly more time in the center area compared to the vehicle group (Fig. 3, *F*–*H*). The open field test suggests that CC treatment also reduces anxiety of the AD mice.

**Figure 4:**
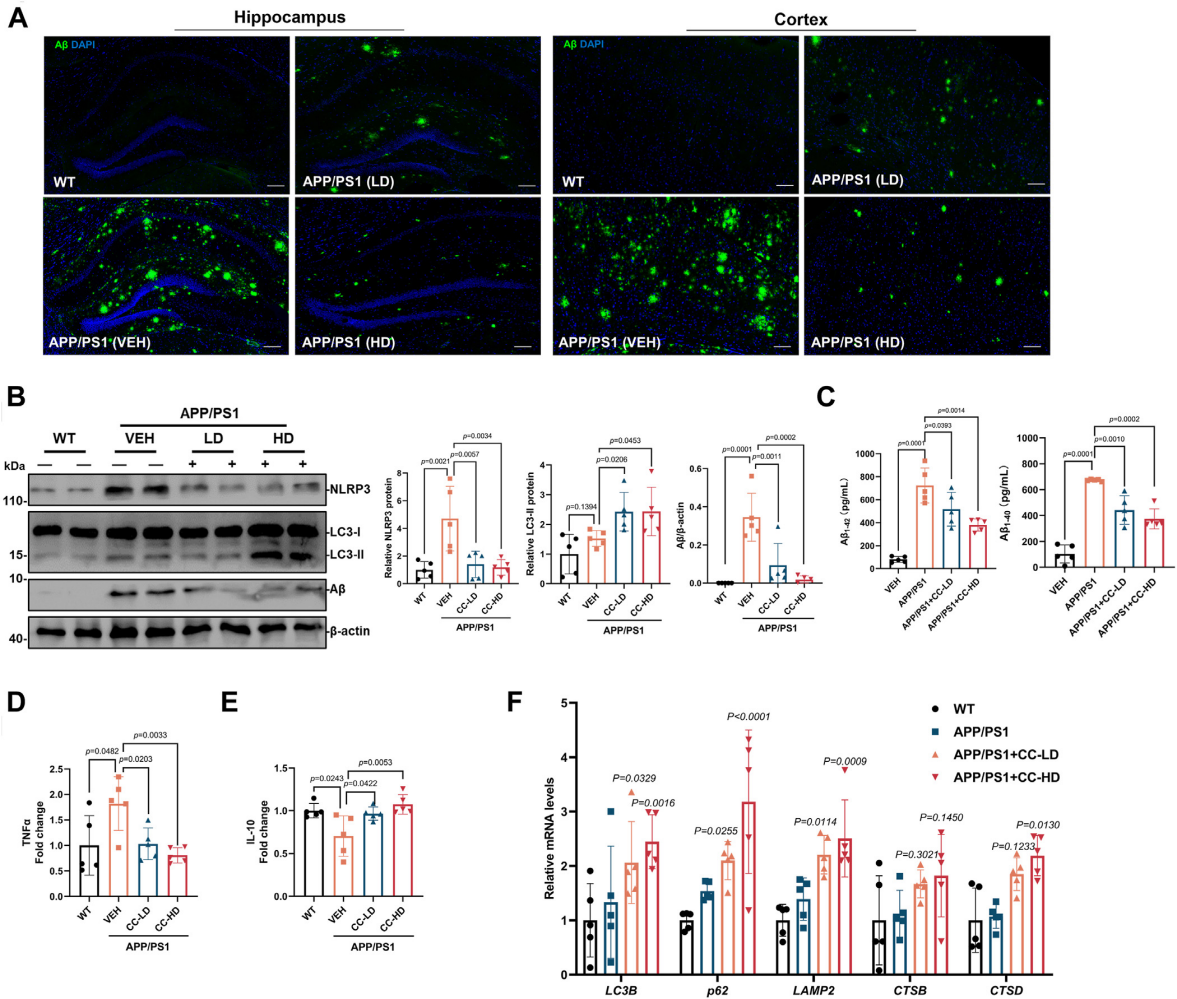
**CC treatment of 7-month-old APP/PS1 AD mice promotes clearance of Aβ plaques and improves Alzheimer's disease symptoms**. *A*, representative images of the hippocampus and cortex in mouse brain sections immunostained with an antibody against Aβ and DAPI. The scale bar represents 50 μm. *B*, mouse brains were extracted and used for Western blot analysis using antibodies against Aβ, LC3 and NLRP3, β-actin was used as a loading control. *C*, the Aβ_1-42_ and Aβ_1-40_ contents in the mice brain tissues were detected using ELISA. The detected protein levels were quantified by densitometric analysis and are represented as mean band intensity normalized to β-actin. The levels of the cytokines including TNF-α (*D*) and IL-10 (*E*) in the brain tissues were detected using ELISA. *F*, the mRNA levels of genes encoding LC3B, p62, LAMP2, CTSB, and CTSD in the brain tissue were detected by qRT-PCR analysis (n = 5). Data are presented as mean ± SD of three independent experiments. Aβ, amyloid-β; AD, Alzheimer’s disease; APP, Aβ precursor protein; CC, clomiphene citrate; CTSB, cathepsin B; CTSD, cathepsin B; DAPI, 4′,6-diamidino-2-phenylindole; LC3, microtubule-associated protein light chain-3; NLRP3, NOD-like receptor family pyrin domain-containing 3; qRT-PCR, quantitative reverse transcription PCR; TNF-α, tumor necrosis factor-alpha.

Additional changes in Aβ pathology, autophagy, and inflammatory response were also determined in the CC-treated mice. Immunofluorescence staining of the brain sections showed that the size of Aβ plaques was significantly reduced in the CC-treated AD mice (Fig. 4*A*), and further Western blot analysis showed that the protein level of Aβ was also significantly decreased in brain tissues of CC-treated AD mice (Fig. 4*B*). In addition, the Aβ_1-42_ and Aβ_1-40_ contents in the brain tissue were measured using ELISA and discovered that both Aβ_1-42_ and Aβ_1-40_ levels in the AD mice were reduced after CC treatment (Fig. 4*C*). The protein levels of LC3-II were significantly increased in brain tissues of CC treatment AD mice (Fig. 4*B*), indicating activation of autophagy. Analysis of neuroinflammatory marker proteins also indicated that 12-month-old APP/PS1 mice contained higher levels of NOD-like receptor family pyrin domain-containing 3 (NLRP3) and the proinflammatory cytokine tumor necrosis factor-alpha (TNF-α) in the brain compared to the WT mice, while CC treatment obviously decreased the protein levels of NLRP3 and TNF-α in the AD mice (Fig. 4, *B* and *D*). In contrast, the interleukin-10 (IL-10) antiinflammatory cytokine was increased after CC treatment (Fig. 4*E*). These results indicate that CC reduces neuroinflammation in the brain of AD mice. In addition, in order to determine whether the CC treatment was also directly involved in reducing inflammatory activation other than through clearing Aβ aggregates, primary microglia cells were treated with lipopolysaccharides (LPS) in the presence of CC and showed that CC treatment significantly reduced the LPS-induced increasing protein level of NLRP3 (Fig. S3), which suggests CC not only promotes clearance of Aβ aggregates but is also directly involved in reducing neuroinflammation in AD.

In addition, to verify the effect of CC on the transcriptional activity of TFEB, qRT-PCR was performed using the brain samples, and demonstrated that the CC treatment upregulated the expression of TFEB downstream genes encoding LC3B, p62, LAMP2, CTSB, and CTSD, which are associated with autophagy and lysosomal function (Fig. 4*F*). Collectively, these results suggest that CC activates the autophagy-lysosome pathway and contributes to improving the AD symptoms in mice.

### Clomiphene citrate treatment of 3-month-old APP/PS1 mice reduces Aβ and improves Alzheimer's disease symptoms

**Figure 5:**
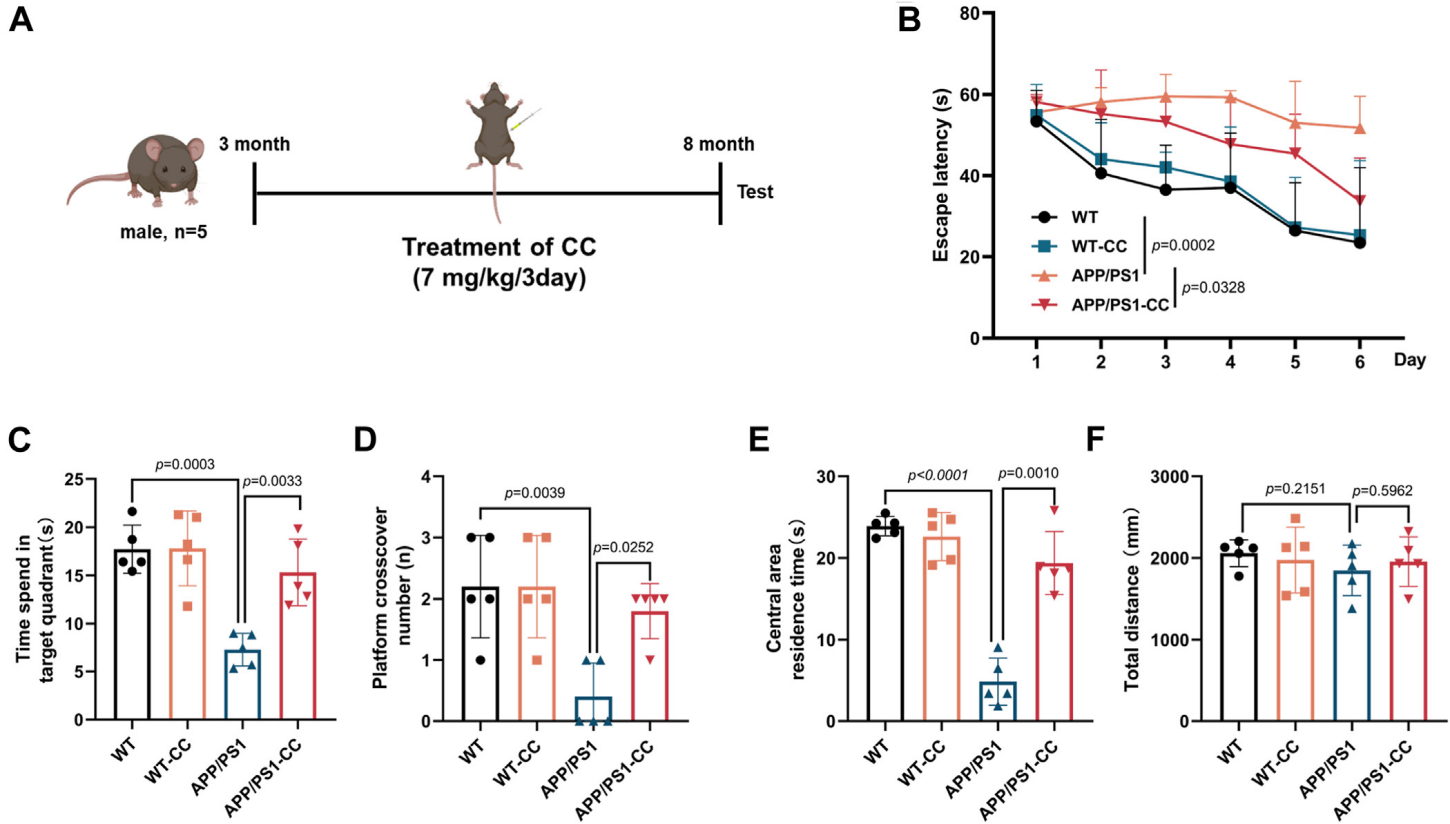
**CC treatment of 3-month-old APP/PS1 AD mice ameliorates cognitive decline. The Morris water maze (MWM) and open field test were used for behavioral test**s. *A*, schedule of animal treatments and experimental protocols. Three-month-old male APP/PS1mice or C57BL/6 mice (WT) were administered intraperitoneally with CC (7 mg/kg body weight) or vehicle (0.9% normal saline) every 3 days up to 8 months of age before testing. n = 5. *B*, latency to escape to a hidden platform during a 6-day MWM training trial period. Further MWM probe trial analysis, time spent in the target quadrant (*C*), and number of times that mice passed through the platform location (*D*). Quantification of the open field test for time stayed in the center area (*E*), and total distance traveled in the open field (*F*). Data are presented as mean ± SD of three independent experiments. AD, Alzheimer’s disease; APP, Aβ precursor protein; CC, clomiphene citrate.

To further address the effect of early treatment of CC on Alzheimer's disease symptoms and Aβ pathology, 3-month-old APP/PS1 mice were treated with CC (7 mg/kg) every 3 days to 8 months of age (Fig. 5*A*). Behavioral tests were then performed. During training trials using the MWM test, CC-treated AD mice took less time to locate the hidden platform (Fig. 5*B*) compared to the vehicle control. During the probe trial in MWM test, CC-treated AD mice spent more time in the target quadrant (Fig. 5*C*) and crossed the platform significantly more often (Fig. 5*D*). Consistent with previous results of CC treated 7-month-old APP/PS1 mice, the MWM test indicated that early treatment of CC improved progressive cognitive decline of the AD mice. In addition, an open field test showed that CC treated AD mice spent more time in the center area compared to the vehicle group (Fig. 5, *E* and *F*), suggesting that CC treatment reduced anxiety of the AD mice.

**Figure 6:**
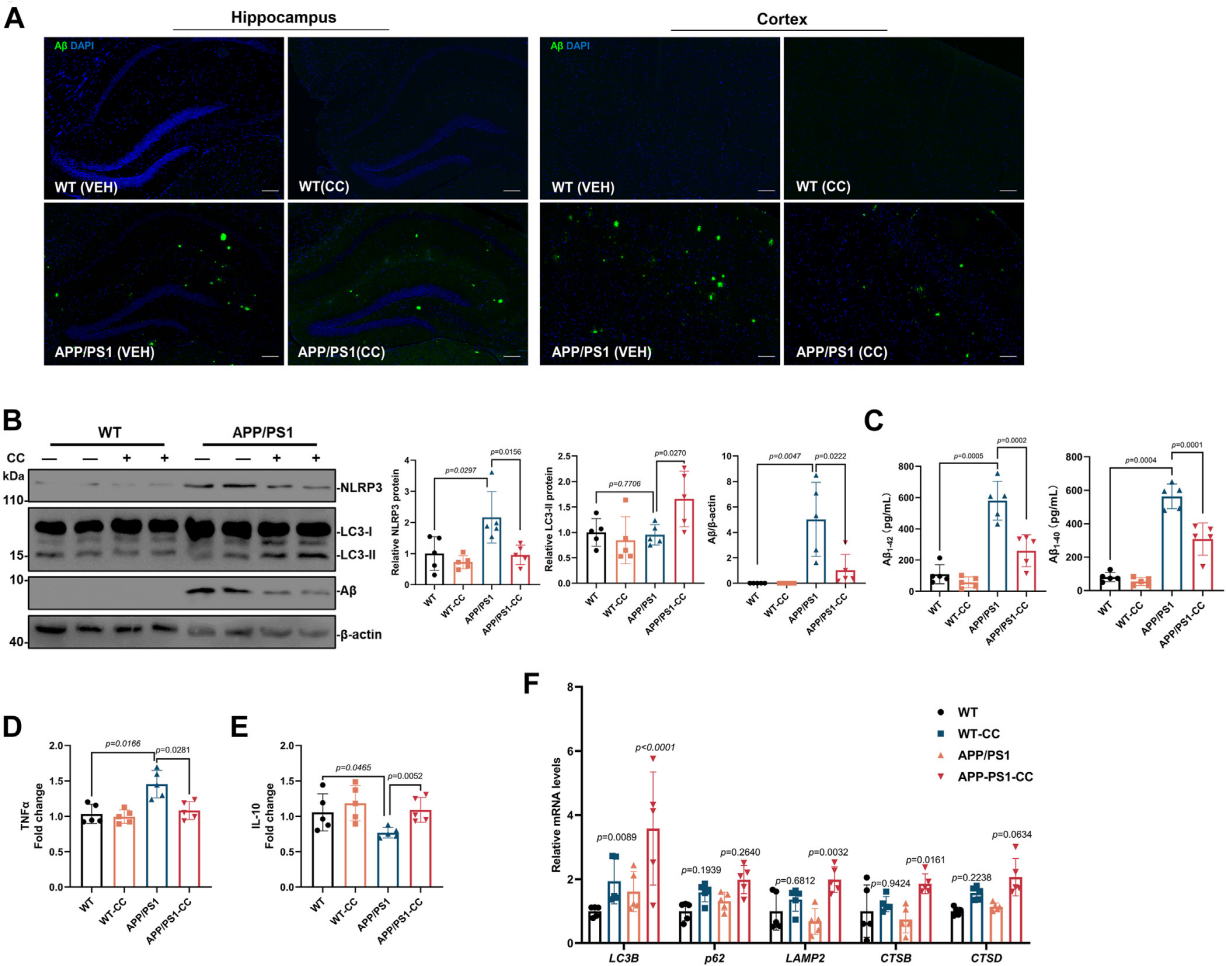
**CC treatment of 3-month-old APP/PS1 AD mice reduces Aβ and improves Alzheimer's disease symptoms**. *A*, representative images of the hippocampus and cortex in mice brain sections immunostained with antibody against Aβ and DAPI. The scale bar represents 50 μm. *B*, mouse brains were extracted and used for Western blot analysis using antibodies against Aβ, LC3, and NLRP3, β-actin was used as a loading control. *C*, the protein levels of Aβ_1-42_ and Aβ_1-40_ in mice brain were detected using ELISA. The detected protein levels were quantified by densitometric analysis and are represented as mean band intensities normalized to β-actin. The levels of the cytokines including TNF-α (*D*) and IL-10 (*E*) in the brain tissues were detected using ELISA. *F*, The mRNA levels of genes encoding LC3B, p62, LAMP2, CTSB, f4and CTSD in brain tissue were detected by quantitative reverse transcription PCR analysis (n = 5). Data are presented as mean ± SD of three independent experiments. Aβ, amyloid-β; AD, Alzheimer’s disease; APP, Aβ precursor protein; CC, clomiphene citrate; CTSB, cathepsin B; CTSD, cathepsin B; DAPI, 4′,6-diamidino-2-phenylindole; LC3, microtubule-associated protein light chain-3; NLRP3, NOD-like receptor family pyrin domain-containing 3; TNF-α, tumor necrosis factor-alpha.

Other changes in Aβ pathology, autophagy, and inflammatory response were next determined. Immunofluorescence staining of brain sections showed that the Aβ plaques were reduced in the CC-treated AD mice (Fig. 6*A*), and further Western blot analysis showed that the protein level of Aβ was significantly decreased in brain tissues following CC treatment (Fig. 6*B*). Further ELISA assay showed that Aβ_1-42_ and Aβ_1-40_ levels in the brain of AD mice were reduced after CC treatment (Fig. 6*C*). The protein levels of LC3-II were significantly increased in brain tissues of CC-treated AD mice (Fig. 6*B*), indicating activation of autophagy. We detected the neuroinflammatory marker proteins in the brain and found that CC treatment decreased the protein levels of NLRP3 and TNF-α in the AD mice (Fig. 6, *B* and *D*). The IL-10 antiinflammatory cytokine was increased after CC treatment (Fig. 6*E*). These results indicate that CC treatment relieves neuroinflammation in the brain of AD mice. To further verify the effect of CC on the transcriptional activity of TFEB, qRT-PCR was performed using the brain samples, which showed that CC upregulated the expression of TFEB downstream genes encoding LC3B, p62, LAMP2, CTSB, and CTSD, which are associated with autophagy and lysosomal biogenesis (Fig. 6*F*). These results suggest that early treatment of CC improves the AD symptoms by activating the autophagy-lysosome pathway.

### Clomiphene citrate induced-nuclear translocation of TFEB is regulated by acetylation

**Figure 7:**
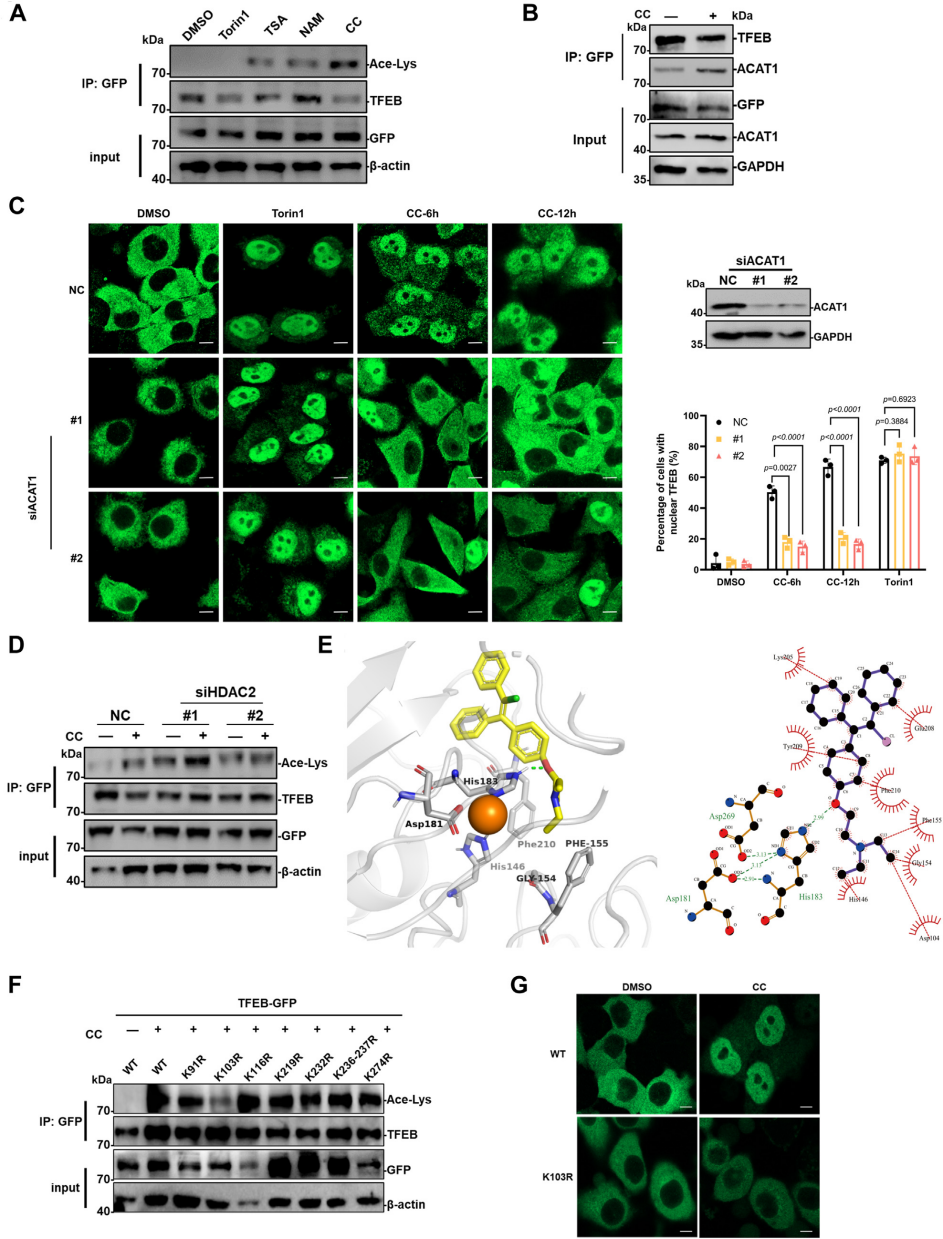
**CC treatment promotes acetylation of TFEB and its nuclear translocation**. *A*, HeLa cells stably expressing a TFEB-GFP were treated with 10 μM CC, 10 μM TSA, 4 mM NAM for 24 h, and 250 nM Torin1 for 6 h, cells were extracted and immuno-precipitated using antibody against GFP, then analyzed by Western blot using acetylated-lysine Mouse mAb (Ace-Lys) and antibodies against TFEB, GFP, β-actin. *B*, HeLa cells stably expressing a TFEB-GFP were treated with or without 10 μM CC for 24 h, cells were extracted and immunoprecipitated using antibody against GFP, then analyzed by Western blot using antibodies against TFEB, ACAT1, GFP, and GAPDH. *C*, HeLa cells stably expressing TFEB-GFP were transfected with two siRNAs targeting ACAT1, 3 days after transfection, 10 μM CC was added and further treated for 6, 12 h, then observed using confocal fluorescence microscopy. Torin1 (200 nM, 1 h) was used as a positive control. The percentage of cells with nuclear TFEB-GFP fluorescence was quantified. The scale bar represnts 10 μm. *D*, HeLa cells stably expressing TFEB-GFP were transfected with two siRNAs targeting ACAT1, 3 days after transfection, 0 or 10 μM CC was added and further treated for 12 h. Cells were extracted and immunoprecipitated using antibody against GFP, then analyzed by Western blot using acetylated-lysine Mouse mAb (Ace-Lys) and antibodies against TFEB, GFP, β-actin. *E*, the molecular docking analysis of En-cc with HDAC2 using AutoDock version 4.2 software (https://autodock.scripps.edu/download-autodock4). The binding modes were visualized in both three- and two-dimensional representations using PyMOL software (version 3.0.0) and Ligplot software (version 2.2.8), respectively. The zinc-binding active sites of HDAC2 were shown as His146, Gly154, Phe155, Asp181, His183, and Phe210. *Orange ball* represents the zinc ion. Interaction plot by LIGPLOT (*right*) (*Dotted red* and *dotted green lines* represent hydrophobic contacts and hydrogen bonds with its length, respectively). *F*, HeLa cells stably expressing a TFEB-GFP (WT) and a series of mutants (KR) were generated and treated with 10 μM CC for 24 h. Cells were extracted and immunoprecipitated using antibody against GFP, then analyzed by Western blot using acetylated-lysine mouse mAb (Ace-Lys) and antibodies against TFEB, GFP, and β-actin. *G*, HeLa cells stably expressing a TFEB-GFP (WT) and mutant (K103R) were treated with or without 10 μM CC for 24 h, then observed using confocal fluorescence microscopy. The scale bar represents 10 μm. ACAT1, acetyl-CoA acetyltransferase 1; CC, clomiphene citrate; En-CC, enclomiphene citrate; HDAC, histone deacetylase; NAM, nicotinamide; TFEB, transcription factor EB; TSA, trichostatin A.

Nuclear translocation of TFEB is regulated by multiple mechanisms, and the best studied of which is mTORC1-mediated phosphorylation at serine 211, which prevents its translocation into the nucleus (24). We have shown that CC induced-nuclear translocation of TFEB is independent of mTORC1 (18). It has been reported that acetylation of TFEB regulates its nuclear translocation in association with acetyl-CoA acetyltransferase 1 (ACAT1) and histone deacetylase 2 (HDAC2) (25). We therefore investigated whether CC induced-nuclear translocation of TFEB was dependent upon acetylation. Firstly, CC along with the pan-HDAC inhibitor trichostatin A (TSA), NAD-dependent protein deacetylase sirtuin1 (SIRT1) inhibitor nicotinamide (NAM) and mTOR1 inhibitor Torin1, were used to treat HeLa cells stably expressing TFEB-GFP. The TFEB-GFP fusion proteins were immunoprecipitated from the cell extracts and used for Western blot analysis using an antibody to detect acetylated-lysine. Significant acetylated TFEB-GFP fusion proteins were detected in the CC-treated cells, the TSA and nicotinamide-treated cells also showed acetylated TFEB-GFP, but not for the Torin 1 treatment (Fig. 7*A*). To further address whether CC is involved in the TFEB-ACAT1 interaction, immunoprecipitation experiments showed that CC treatment enhances the interaction between TFEB and ACAT1(Fig. 7*B*). In addition, knockdown of ACAT1 significantly reduced CC-induced TFEB translocation to the nucleus in the TFEB-GFP stably expressed HeLa cells (Fig. 7*C*), as well as decreased nuclear translocation of endogenous TFEB in HeLa and PC12 cells (Fig. S4, *A* and *B*). These results suggest that ACAT1 is involved in CC-induced acetylation of TFEB and its translocation to the nucleus.

Moreover, knockdown of HDAC2 significantly promoted TFEB acetylation as reported before, demonstrating the importance of HDAC2 in regulation of TFEB acetylation (Fig. 7*D*) (25). Interestingly, CC treatment further increased TFEB acetylation induced by knockdown of HDAC2 (Fig. 7*D*), which suggests CC is involved in the inhibition of HDAC2. Several zinc-dependent HDACs have been reported to be associated with acetylation of TFEB (26, 27), and molecular docking analysis was further used to estimate the interactions between HDACs and CC. The conserved amino acid residues at the active sites of zinc-dependent HDACs were firstly analyzed according to reports in the literature (Table S1) (28, 29, 30). CC is a mixture of two diastereoisomers containing 62% *trans*-isomer of enclomiphene citrate (En-CC) and 38% cis isomer of zuclomiphene citrate (Zu-CC). Molecular docking analysis revealed that En-CC had a much higher binding ability with HDACs than Zu-CC based on the comparation of the binding energy value (ΔG), and HDAC2 had the most stable binding with En-CC, followed by HDAC5 and HDAC6 (Fig. 7*E* and Table 1). A hydrogen bond was formed between En-CC and His183, a conserved active site in HDAC2, with a bond length of 2.99 Å. In addition, En-CC forms a hydrophobic force with His146, Gly154, Phe155, and Phe210 localized at the HDAC2 active pocket for which zinc ion binding. Consistently, TSA showed a general high binding affinity with the deacetylases (Table 1).

**Table 1:** Comparation of the binding ability between En-CC, Zu-CC, TSA and HDACs

	Compounds	HDAC1	HDAC2	HDAC5	HDAC6	HDAC9
Δ G (kcal/mol)	En-CC	−3.39^a^	−5.68^b^	−4.78^c^	−4.51^c^	−3.62^a^
	Zu-CC	−3.19^a^	−3.85^a^	−3.56^a^	−3.26^a^	−3.02^a^
	TSA	−4.86^c^	−5.78^b^	−4.61^c^	−4.67^c^	−4.49^c^

The combined mode was visualized using PyMOL (version 3.0.0) and Ligplot software (version 2.8.8). The final binding energy value (Δ G) for sorting and analysis.

a Indicating binding ability, −3 > Δ G > −4.

b Indicating very strong binding ability, Δ G < −5.

c Indicating strong binding ability, −4 > Δ G > −5.

To further identify the CC induced-acetylation sites on TFEB, a series of mutants were generated by mutating lysine (K) to arginine (R), and used to generate stable cell lines expressing these mutated TFEB-GFP proteins. Immunoprecipitation and Western blot analysis showed that K103R mutation significantly inhibited the CC-induced acetylation of TFEB (Fig. 7*F*). Fluorescence microscopy also indicated that K103R mutation significantly inhibited the CC-induced nuclear translocation of TFEB (Figs. 7*G* and S4, *C* and *D*). These results suggest that CC is a potential inhibitor of zinc-dependent HDACs, and CC promotes nuclear translocation of TFEB by stabilizing its acetylation modified by ACAT1.

## Discussion

Though AD pathogenesis is still not fully understood, the defining pathological features of AD includes aggregation of Aβ and tau proteins and neuroinflammation (31). Autophagy is a major intracellular process to clear protein aggregates and the mammalian nervous system depends heavily on autophagy to clear large and insoluble protein aggregates to maintain protein homeostasis (32). Recent evidence has indicated that dysfunctional autophagy contributes to AD pathogenesis; therefore, the autophagy-stimulating strategies for AD therapeutics have been investigated and reported (33). For example, nilotinib, an FDA-approved drug for leukemia, induced autophagy *via* mTOR inhibition, and reduced amyloid plaques and improved cognition in AD model Tg-APP mice (34). A clinical trial study indicated that nilotinib treatment significantly reduced amyloid burden in the frontal lobe, and cerebrospinal fluid Aβ was also decreased (35). In this study, 7- and 3-month-old APP/PS1 mice were treated with CC, and both treatments showed significant reduction of Aβ aggregation and Aβ protein level, which leads to neuroprotection and improvement of cognitive function. The transgenic APP/PS1 mice are born destined to develop AD symptoms, although Aβ deposition begins at 3 to 4 months of age in the hippocampus (20), while cognition is impaired at 7 months (21). CC treated 3-month-old APP/PS1 mice delayed developing AD symptoms, which suggests early treatment of AD using CC is a potential strategy to prevent AD by stimulating autophagy.

Activation of inflammation in the brain is one of the pathogenic hallmarks of AD. Autophagy also has unique roles in regulating the inflammatory response in both the innate and adaptive immune system (36). The NLRP3 inflammasome is a key molecular mechanism in AD neuroinflammation, the Aβ and tau aggregates can lead to the assembly and activation of NLRP3 inflammasome in microglia and astrocytes in the brain (37). Autophagic removal of NLRP3 inflammasome components, activators, and cytokines can reduce inflammasome activation and the inflammatory response (38). Here, we showed that CC treatment improved the neuroinflammation in the brain of AD mice by reducing the levels of NLRP3 and TNF-α. Interestingly, CC treatment also reduced the LPS-induced increasing NLRP3 in primary microglia cells, suggesting CC is directly involved in mitigating neuroinflammation in AD.

TFEB is a master transcriptional regulator of autophagy and lysosome biogenesis that promotes intracellular clearance of pathogenic factors in many diseases including neurodegenerative diseases. Therefore, TFEB has been given attention as a drug target and TFEB agonists have been identified and studied. Though the mTOR1 inhibitors, such as Torin1 and rapamycin, induce a strong activation of TFEB, inhibiting mTORC1 is not an ideal strategy to activate TFEB due to its other important functions on cell growth and protein synthesis (39). Therefore, studies on the TFEB agonists independent of mTORC1 have been performed (40). Here, we show that CC promotes nuclear translocation of TFEB by regulating its acetylation. Acetylation plays crucial roles in the regulation of autophagy by targeting core components of the autophagy machinery (41). It has been reported that acetylation of TFEB leads to its translocation from the cytosol to the nucleus (25, 26), with acetyltransferases including ACAT1 and HDACs involved in the translocation process. Another study showed that acetyltransferase GCN5 targets TFEB in the nucleus, with GCN5-mediated acetylation at K274 and K279 disrupting the dimerization of nuclear TFEB and inhibiting its binding to promoters of target genes (42). Whereas, SIRT1 was reported to interact with nuclear TFEB and deacetylated TFEB at K116 to enhance its transcriptional activation (43). These studies indicate that modulation of TFEB by acetylation occurs in a localization and modification site-dependent manner, likely to balance TFEB-mediated activation of autophagy and lysosome biogenesis under different physiological conditions. ACAT1 is one of the enzymes that catalyzes the final step of the mitochondrial beta-oxidation pathway by breaking down fatty acids into acetyl-CoA. Recently, report indicates that ACAT1-mediated fatty acid oxidation is associated with increased histone acetylation, and the levels of ACAT1 and histone acetylation are abnormally elevated in patients with obesity (44). Lysine acetyltransferase ACAT1 and deacetylase HDAC2 control the TFEB acetylation modification, and treatment of HDACs inhibitor suberoylanilide hydroxamic acid recruits more ACAT1 to TFEB for acetylation (25). Here, we showed CC is a HDACs inhibitor and treatment of CC increases the interaction between ACAT1 and TFEB. Further study is required to clarify how ACAT1 and HDACs coordinate to keep the balance of acetylation modification on TFEB under different physiological and pathological conditions.

CC was first approved by the FDA in 1967 and has been widely used in women for induction of ovulation. The daily dose of CC in humans is 50-100 mg, equally about 7 to 14 mg/kg/day in mice. The acute oral median lethal dose (LD_50_) of CC is 1700 mg/kg in mice, the biological half-life of CC is 5 to 7 days, and has a good property of crossing the blood–brain barrier (https://www.drugbank.ca/drugs/DB00882). In this study, the mice were administered intraperitoneally with 7 or 14 mg/kg CC, once every 3 days rather than daily, in order to ensure the administration of CC was effective but also safe. CC treatment indeed induced activation of TFEB and autophagy in both WT and AD mice, and months of treatment with CC did not show clear toxicity, assessed by measuring bodyweight. For the future work, it will be interesting to find out whether oral administration, and extending administration time of CC, such as once a week, still can achieve the same effect on AD mice, as once weekly medical regimen will particularly benefit the AD patients compared to medicating daily.

Though CC is an estrogen receptor modulator, it has both estrogenic and antiestrogenic properties. In addition to being widely used in women for induction of ovulation, CC has also been used off-label to treat secondary male hypogonadism by raising testosterone levels (45). It has been reported that estrogen plays a protective role for AD, particularly in postmenopausal women (46, 47). Our previous study indicates that CC activates TFEB-mediated autophagy and lysosomal biogenesis independent of estrogen receptor pathway (18). Here, we showed that CC treatment significantly decreased the Aβ level in the primary microglia cells, as well as in the brain of male APP/PS1 mice, which contributes to ameliorating AD symptoms. We found that CC is a potential inhibitor of HDACs with the most inhibition on HDAC2. It has been reported that the pan-HDAC inhibitor TSA reduced Aβ plaques and improved the memory of APP/PS1 mice by inducing acetylation of TFEB and promoting lysosome biogenesis (26). These studies suggest the potential role of CC in modulating AD-related phenotypes as a TFEB agonist through inhibiting HDACs. Our animal experiments were done in male APP/PS1 mice; it is worth for the future subsequent study to add female AD mice to determine if and how CC acts in both sexes.

In summary, using a mouse model of AD, we demonstrate that TFEB agonist CC activates the ALP and ameliorates AD symptoms including Aβ pathology, cognitive function, and inflammation. Our studies support the idea that activation of autophagy is associated to improving health span. Therefore, activation of the TFEB-mediated autophagy-lysosome pathway by TFEB agonist CC can be potentially applied in the treatment or prevention of AD.

## Experimental procedures

### Reagents and antibodies

Unless otherwise stated, all chemicals were purchased from Sigma-Aldrich. CC (purity 99.88%), En-CC(purity 99.05%) and Torin1 (purity 98.77%) were procured from Selleckchem. Zu-CC (purity 98.09%) purchased from MedChemExpress, and dissolved in dimethyl sulfoxide for stock. The anti-LC3B (1:2500, L7543) antibody and anti-GFP (1:5000, SAB2702211) antibody were obtained from Sigma-Aldrich. Antibodies against Cathepsin B (CTSB) (1:2000, ab190077) and LAMP2 (1:2000, ab25631) were purchased from Abcam. Antibodies against Aβ (1:1000, 8243), acetylated-lysine (1:1000, 9441), NLRP3 (1:1000, 15,101) and TFEB (1:1000, 37,785) were acquired from Cell Signaling Technology. Antibodies against β-actin (1:500, sc-47778) and GAPDH (1:1000, sc-32233) were procured from Santa Cruz Biotechnology. Anti-ACAT1 antibody (1:2000, ER62569) was purchased from HuaBio. The anti-histone H3 antibody (1:1000, KM9005), horseradish peroxidase-conjugated goat anti-rabbit (1:5000, LK2001) and anti-mouse secondary antibodies (1:5000, LK2003) were purchased from Sungene Biotech.

### Cell culture

PC12, SH-SY5Y and HeLa cell lines were obtained from the American Type Culture Collection. The cells were cultured in RPMI-1640 and Dulbecco's modified Eagle's medium/F-12 medium (Gibco), supplemented with 10% fetal bovine serum and 1% antibiotics under a 5% CO2 atmosphere at 37 °C.

### Cell viability assay

The cell viability was assessed using the MTT dye absorbance. Cells were plated in a 96-well plate at a density of 6 × 10³ cells per well. Subsequently, the cells were treated with various concentrations of CC (0–30 μM) for either 24 or 48 h. Following the treatment period, MTT solution (20 μl, 0.5 mg/ml in PBS) was added into each well, and the plates were incubated at 37 °C for 4 h. Dimethyl sulfoxide (200 μl) was then added to dissolve the formed crystals. The wells were gently shaken to ensure complete dissolution, and the absorbance of each well was measured at a wavelength of 490 nm using microplate reader.

### Plasmids construction and stable cell lines

The acetylation sites on TFEB were mutated by site-directed mutagenesis. The full-length human TFEB gene and mutants were cloned into pLVX-AcGFP-N1 lentiviral vector using the EcoRI and BamHI cloning sites. All constructs were verified by DNA sequencing. The lentivirus particles were generated by transfecting HEK-293T cells with pMD2.G and psPAX2 packaging plasmids and the corresponding backbone plasmids using Lipofectamine 2000 (Invitrogen) according to the manufacturer’s instructions. Viruses were collected 48 h posttransfection, and used for infection overnight in the presence of 8 μg/ml polybrene. HeLa cells were infected with recombinant lentiviruses and selected in 1 μg/ml puromycin containing medium for at least 2 weeks. The positive clones were picked into 96 well plates for further culture to obtain TFEB-GFP and mutants stably expressed cell lines. Plasmids expressing TFEB short hairpin RNA (shRNA) (5′CCGGCCCACTTTGGTGCTAATAGCTCTCGAGAGCTATTAGCACCAAAGTGGGTTTTTG3′) was constructed using pLKO.1-puro lentiviral vector. PC12 cells were infected with the recombinant lentiviruses and selected with 1 μg/ml puromycin.

### Primary microglia isolation

Newborn mice within 3 days of birth were sterilized with 75% ethanol, the brain tissue was taken out after carefully removing the meninges and blood vessels, and the hippocampal tissue peeled off. Briefly, 0.125% trypsin was added and digested 10 min at 37 °C, before adding an equal volume of Dulbecco's modified Eagle's medium culture solution containing 10% fetal bovine serum to terminate the digestion. After filtering the cell suspension through a 200 Mesh cell sieve to remove the undigested tissue block, cells were cultured at 37 °C until a fused glial cell layer was formed.

### Small interfering RNA (siRNA)

siRNAs targeting ACAT1, HDAC2 were purchased from GenePharma and transfected using lipofectamine 2000 (Invitrogen, 11668019) according to the manufacturer's protocol. The siRNAs used are ACAT1 #1 (5′GGAACGGAGUUAUGUAUCATT3′), ACAT1 #2 (5′ CAUGGGUAAUGUUCUACAATT 3′), HDAC2 #1 (5′CCAUGAAGCCUCAUAGAAUTT3′), HDAC2 #2 (5′ GCAGAUGCAGAGAUUUAAUTT 3′), and the negative control (5′ UUCUCCGAACGUGUCACGUTT 3′).

### Immunofluorescence

Cells were cultured on polylysine-coated glass coverslips. After CC treatment, cells were fixed with 4% paraformaldehyde and permeabilized with 0.1% Triton X-100 in PBS. Then blocked with 3% bovine serum albumin (BSA) for 30 min at room temperature. The cells were incubated with primary antibody overnight at 4 °C and then with Alexa fluor 488 labeled secondary antibody for 2 h at room temperature. Coverslips were sealed with Mowiol, and the cells were visualized and imaged using a fast super-resolution laser confocal microscopy system (Zeiss Lsm 980 airyscan2).

### Western blot

Prepare lysis buffer RIPA (50 mM Tris–HCl pH 7.4, 150 mM NaCl, 0.1% SDS, 1% Triton X-100, and 1% sodium deoxycholate) containing a mixture of protease inhibitors (MCE). For extraction of proteins from cells, cultured cells were collected, an appropriate volume of RIPA lysis buffer (containing proteinase inhibitors) was added and lysed on ice for 30 min. The supernatant was collected after centrifugation at 12,000 rpm for 10 min at 4 °C. For extraction of proteins from tissues, the tissue samples were washed twice with cold PBS, cut into small pieces and put into a mortar. Liquid nitrogen was added, and the tissue samples were rolled and ground into powder with a pestle. RIPA lysis buffer was added according to the volume ratio of 1:10, homogenized on ice using a glass homogenizer for 30 min, and the supernatant was collected by centrifugation (14,000 rpm, 4 °C, 10 min). SDS loading buffer was added to the protein samples for SDS-PAGE electrophoresis, and then transferred to polyvinylidene fluoride membranes (Roche). Polyvinylidene fluoride membranes were firstly blocked with 5% skim milk (in PBST) for 1 h, before being incubated with specific primary antibody overnight at 4 °C. Subsequently, it isr washed with PBST, incubated with appropriately coupled horseradish peroxidase-labeled secondary antibody for 2 h at room temperature, and finally washed with PBST and PBS. Immunoblotting was conducted using chemiluminescent reagents, and visualization was performed using a chemiluminescent detection system (GE HealthCare). Quantitative analysis was conducted using the ImageJ software (https://imagej.net/software/imagej2, Wayne Rasband, NIH Bethesda, MD USA), with the β-actin protein level serving as a loading control.

### Immunoprecipitation

Cells were collected and added with prechilled NP-40 lysis buffer (containing proteinase inhibitors), incubated the lysates on ice for 30 min after centrifugation (12,000 rpm, 4 °C, and 10 min), the supernatant was collected and incubated with 5% BSA-pretreated camel anti-GFP antibody-conjugated agarose beads (V-nanoab Biotechnology) for 2 h at 4 °C with agitation. The supernatant was discarded after centrifugation (2500*g*, 4 °C, 3 min), then the beads were washed with lysis buffer. Subsequently, SDS sample buffer was added and heated at 95 °C for 10 min. The protein samples were collected after centrifugation (2500*g*, 4 °C, for 3 min), followed by immunoblotting analysis.

### RNA extraction and qRT-PCR

Total RNA was extracted from cells or mouse tissues using the TRIzol Reagent (Life Technologies), and reverse transcription was performed using the All-in-One First-Strand complementary DNA Synthesis SuperMix kit for RT-PCR (TransScript). mRNA levels were detected using quantitative real-time PCR analysis with SybrGreen qPCR Mastermix (DBI Bioscience). The reaction was carried out at 95 °C for 2 min, followed by 40 cycles of 95 °C for 10 s, 60 °C for 30 s, 72 °C for 30 s and finally 95 °C for 1 min. The sequences of primers used for RT-PCR were shown in Table S2.

### Treatment of mice

The ethical approval for the animal experiments conducted in this study was granted by the Ethics Committee of the Tianjin University of Science and Technology, Tianjin, China (2021-Shengwu-002). Animal experiments strictly adhered to national and institutional guidelines for ethical care and use. The APP/PS1 mouse strain (stock No. 004462) was purchased from Jackson Laboratory, USA. For the experiments, both APP/PS1 mice and their WT littermates were used. APP/PS1 mice received intraperitoneal injections of CC (low dose [LD] of 7 mg/kg/3 day, high dose [HD] of 14 mg/kg/3 day) from 7 months of age (already present of Aβ plaques) until 12 months. For another experiment, APP/PS1 mice received CC (7 mg/kg/3 day) from 3 months of age (prior to Aβ plaque formation) until 8 months. For intermittent fasting treatment, APP/PS1 mice underwent a 24 h fasting weekly from 3 months of age to 11 months. Behavioral assessments and molecular biology tests were conducted at the end of the treatments.

### Morris water maze

A transparent platform with a diameter of 10 cm was placed in a fixed position 1 cm below the water surface. The mice were trained for six consecutive days and tested 4 times a day, with an average test interval of 90 min. Each trial lasted for 60 s or until the mice found the platform. After each trial, the mice were placed on the platform for 30 s. On day 7, the platform was removed for a detection test lasting 60 s. The software was used to track the animal behavior by video and automatically calculate the behavioral parameters (escape latency, thigmotaxis duration, swimming speed, travel distance, and so on) to analyze and evaluate the effect of treatments on the cognition of AD mice.

### Open field test

The open field test was conducted to evaluate the exploratory behavior of mice in the new environment. The experiment was conducted 24 h after the last administration. The mice were placed in the center of the open space (25 cm × 25 cm) and explored the environment without any external interference for 5 min. Software is used to measure parameters such as motion mode, dwell time in the center and edge, moving speed, and travel distance.

### Immunohistochemistry

Mouse brain tissues were fixed in 4% paraformaldehyde, paraffin embedded, and pathologically sectioned. The sections were blocked in PBS containing 3% BSA for 1 h, then incubated with Aβ antibody diluted in 3% BSA at 4 °C overnight. After rewarming, the sections were incubated with Alexa fluor 488 labeled secondary antibody at room temperature for 2 h, then the nucleus was stained using 4′,6-diamidino-2-phenylindole (DAPI) for 15 min, and finally sealed with antifluorescence quenched blocking agent. Scanned the sections with a tissue slice digital scanner (Pannoramic 250FLASH), and observed and intercepted the tissue pictures with CaseViewer2.4 software (https://www.3dhistech.com/solutions/caseviewer).

### Enzyme-linked immunosorbent assay (ELISA) for measuring Aβ and inflammatory cytokines

Aβ levels were quantified using commercial ELISA kits against Aβ_1-40_ (cat# MU30299, BIOSWAMP) and Aβ_1-42_ (cat# MU30114, BIOSWAMP) according to the manufacturer’s protocols. The TNF-α (cat# ZC-39024), interleukin 10 (IL-10, cat# ZC-37962) ELISA kits were obtained from ZCIBIO Technology.

### Molecular docking analysis

Free energy binding estimation was performed using AutoDock version 4.2 software (https://autodock.scripps.edu/download-autodock4) and standard protocols. The 3D structures of HDAC1(code 4bkx), HDAC2(code 4ly1), HDAC5(code Q9UQL6), HDAC6(code 5edu), and HDAC9(code Q9UKV0) were obtained from the protein data bank and AlphaFold protein structure database (https://alphafold.com), and CC was obtained from PubChem. The PDBQT file of HDACs containing a protein structure with hydrogen in all polar residues was created. All chemical bonds are rotatable. All calculations were performed using Lamarckian genetic algorithm. For docking studies, the global search exhaustiveness was set to 100, and the docking pose prediction of each compound to HDACs was calculated from each energy minimum. The average affinity value of the best posture is taken as the final affinity value.

### Statistical analysis

Data are presented as the mean ± SD. All of the experiments presented were representatives from at least three independent experiments. Statistical significance was analyzed using the unpaired Student *t* test or one-way analysis of variance (ANOVA) using GraphPad Prism version 8.0 (GraphPad Software, https://www.graphpad.com). *p* value < 0.05 was considered significant.

## Data availability

All data are available from the corresponding authors upon reasonable request.

## Supporting information

This article contains supporting information.

## Conflict of interest

The authors declare that they have no conflicts of interest with the contents of this article.
