# Supplementary material for: TFEB agonist clomiphene citrate activates the autophagy-lysosomal pathway and ameliorates Alzheimer's disease symptoms in mice
Source: J Biol Chem. 2024 Oct 24;300(12):107929. doi: 10.1016/j.jbc.2024.107929 (PMC11599454; doi:10.1016/j.jbc.2024.107929)
Supplement: Supporting Information Figures [file mmc1.docx]

**Supporting Information Figure Legends**

**Figure S1. Clomiphene citrate activates the autophagy-lysosome pathway in PC12 and SH-SY5Y cells**. *A,* MTT assay for viability of PC12 or SH-SY5Y cells exposed to 0-30 μM CC for 24 h or 48 h. *B,* Effects of CC on the protein levels of LC3-II. SH-SY5Y cells were treated with 10 μM CC for 12 h or 24 h. The levels of LC3 were measured by Western blot using antibodies against LC3. LC3-II protein level was quantified using ImageJ analysis and represented as the mean band intensity normalized to β-actin. *C,* Following 10 μM CC treatment for 12 h, SH-SY5Y cells were treated with or without 200 nM Baf A1 for an additional 2 h. LC3 protein levels were detected by Western blot. LC3-II protein level was quantified using ImageJ analysis and represented as the mean band intensity normalized to β-actin. *D*, Expression of LAMP2, CTSB in 10 μM CC treated SH-SY5Y cells were measured by Western blot. Quantitative analysis of the immunoblotted proteins was performed using ImageJ and represented as the mean band intensity normalized to β-actin. *E*, The mRNA levels of genes encoding LC3B, p62, LAMP2, CTSB, CTSD in 10 μM CC treated PC12 or SH-SY5Y cells were detected by qRT-PCR analysis. Data are presented as mean ± SD of three independent experiments.

**Figure S2. Expression profile of Aβ at the deferent ages of APP/PS1 mice.** *A*, Representative images of the cortex in brain sections from APP/PS1 or WT mice at different ages (1, 3, 6, 9 and 12 months) immunostained with an antibody against Aβ and DAPI and visualised using fluorescence microscopy. Scale bar, 50 µm. *B*, The Aβ protein levels in the brain of APP/PS1 or WT mice at different ages (1, 3, 6, 9 and 12 months) were measured by Western blot analysis using antibody against Aβ. Aβ protein level was quantified using ImageJ analysis and represented as the mean band intensity normalized to β-actin. Data are presented as mean ± SD of three independent experiments.

**Figure S3. CC reduces the NLRP3 protein level in LPS-treated primary microglia cells**. Primary microglia cells were treated with 0.5 μg/ml LPS in the presence or absence of 10 µM CC for 12 h and analyzed by Western blot using antibodies against NLRP3, β-actin.

**Figure S4. CC induces acetylation of TFEB and its nuclear translocation**. *A*, PC12 cells were transfected with two siRNAs targeting ACAT1, 3 days after transfection, 10 μM CC was added and cells incubated for a further 12 h. Cells were stained with antibody against TFEB, then observed using confocal fluorescence microscopy. Nuclei were stained using DAPI (blue). Scale bar, 20 μm. *B*, HeLa or PC12 cells were transfected with two siRNAs targeting ACAT1, 3 days after transfection, 10 μM CC was added and further treated for 12 h. Subcellular fractionation of cells was performed using the NE-PER Nuclear and Cytoplasmic Extraction Kit (Thermo Scientific, MA, USA). Western blot was used to detect endogenous TFEB protein levels in the nuclear and cytosolic fractions. GAPDH and Histone H3 were used as the loading controls. *C*, HeLa cells stably expressing a TFEB-GFP (WT) and mutants (KR) were treated with 10 µM CC for 24 h, then observed using fluorescence microscopy. Torin1 (200 nM, 1 h) was used as a positive control. Scale bar, 20 μm. *D*, HeLa cells stably expressing a TFEB-GFP (WT) and mutant (K103R) were treated with or without 10 µM CC for 12, 24 h. Subcellular fractionation of cells was performed using the NE-PER Nuclear and Cytoplasmic Extraction Kit (Thermo Scientific, MA, USA). Western blot was used to detect TFEB-GFP protein levels in the nuclear and cytosolic fractions. GAPDH and Histone H3 were used as the loading controls.

**Supporting Information Figures**

**Figure S1**

**
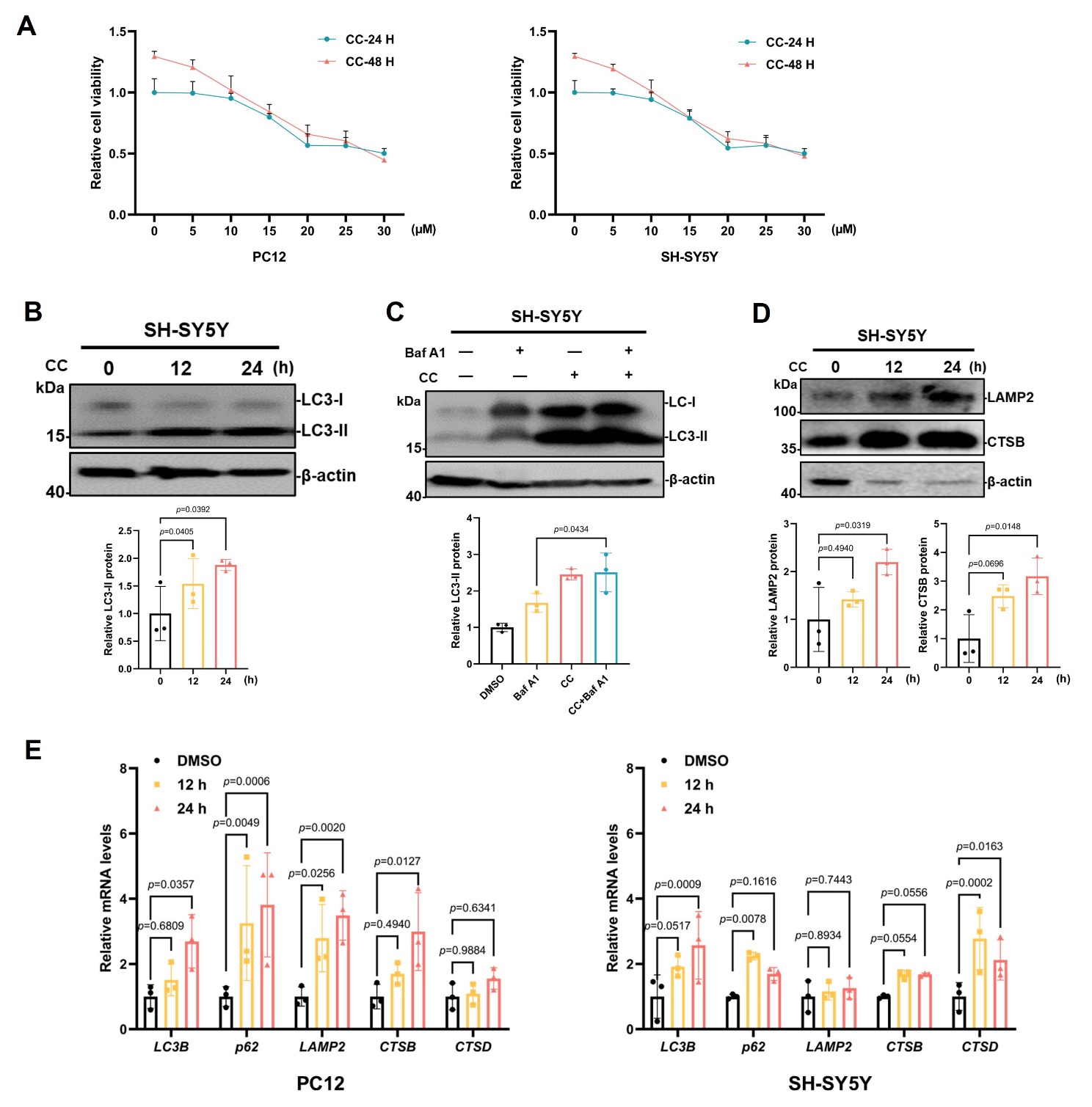
**

**Figure S2**

**
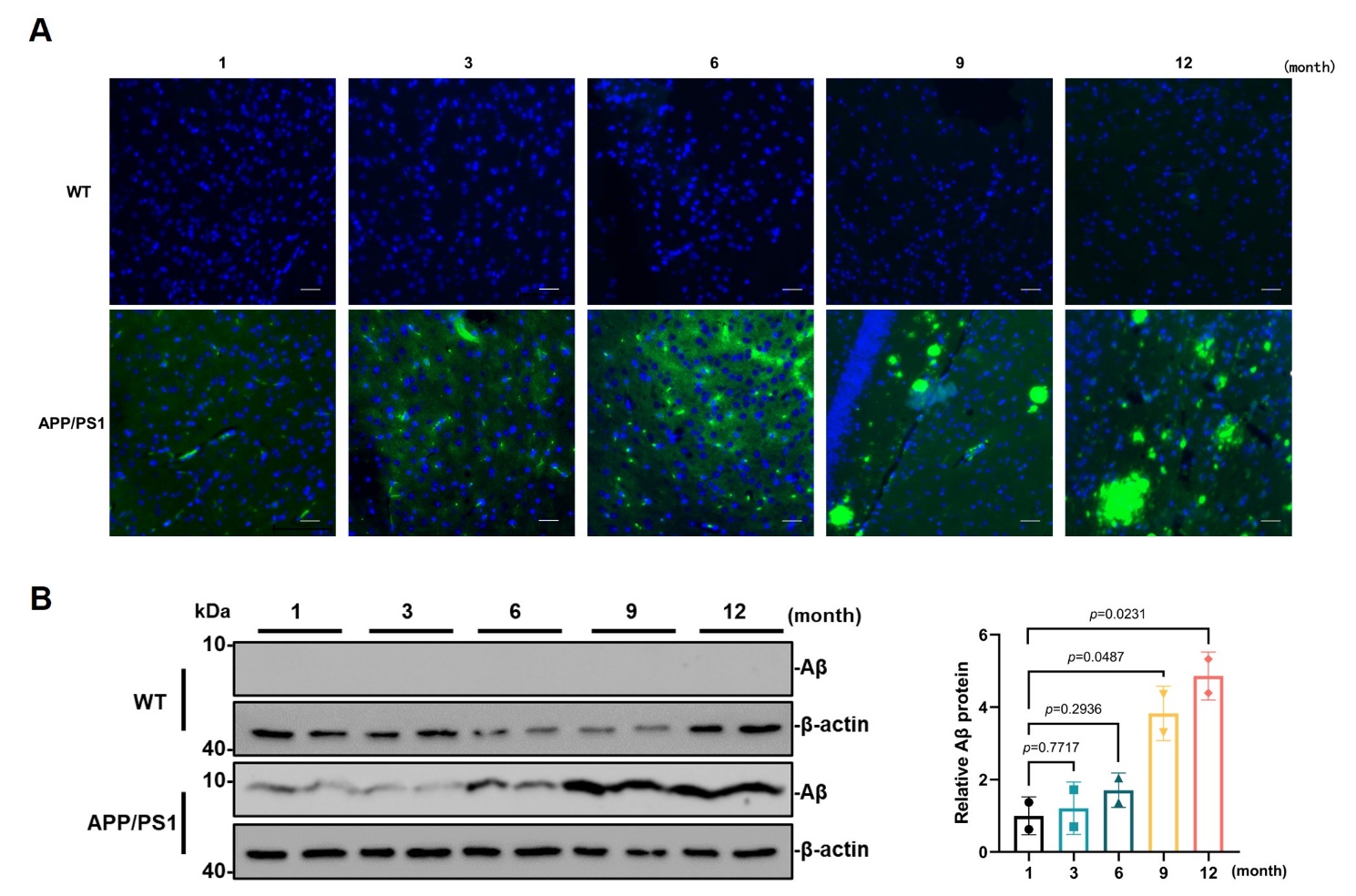
**

**Figure S3**

**
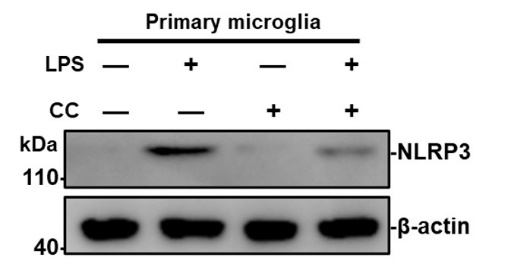
**

**Figure S4**

**
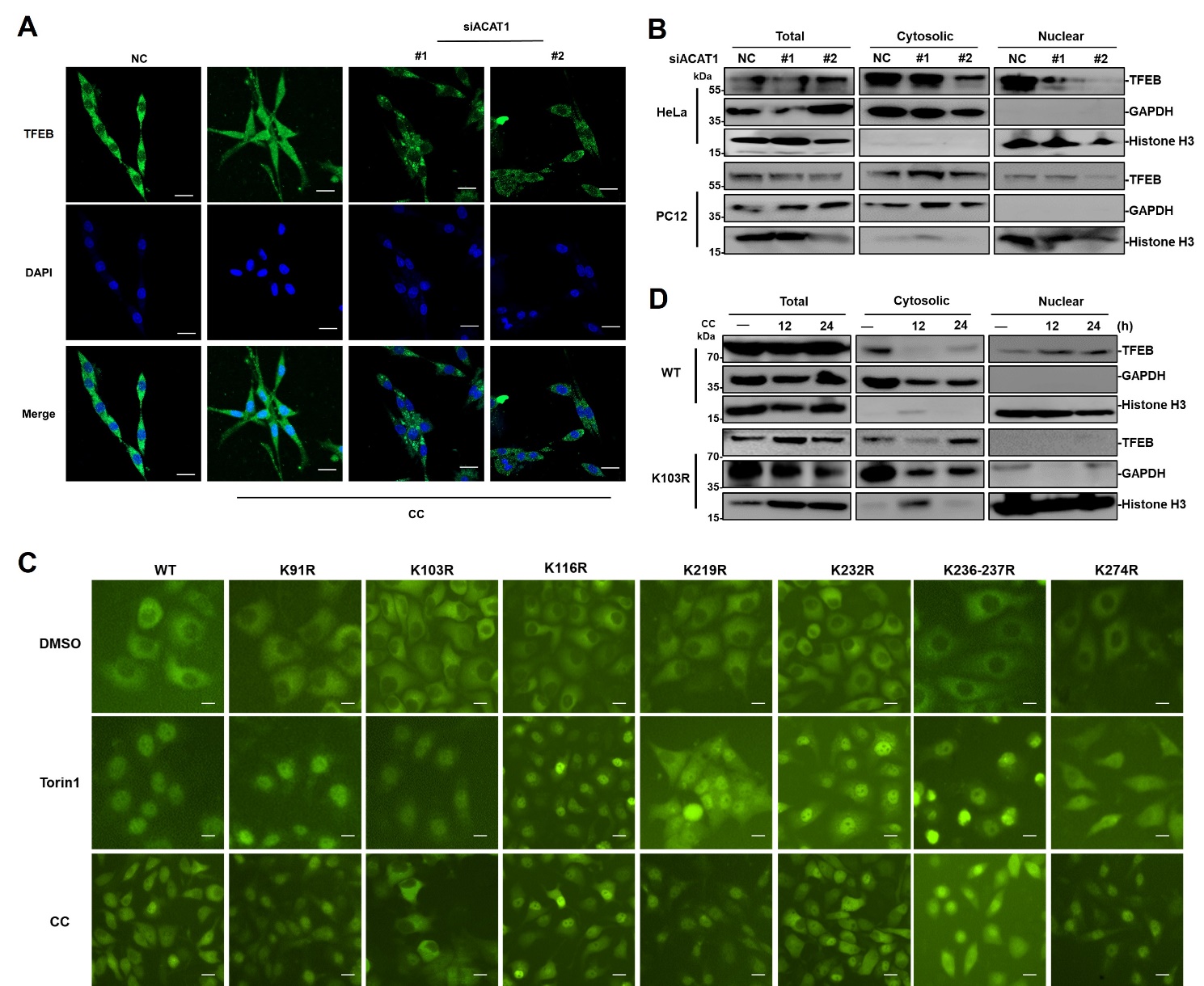
**
