# Supplementary material for: TFEB agonist clomiphene citrate activates the autophagy-lysosomal pathway and ameliorates Alzheimer's disease symptoms in mice
Source: J Biol Chem. 2024 Oct 24;300(12):107929. doi: 10.1016/j.jbc.2024.107929 (PMC11599454; doi:10.1016/j.jbc.2024.107929)
Supplement: Supporting Information Tables [file mmc2.docx]

**Supporting Information Tables**

**Table S1. Conserved amino acid residues at the active sites of human zinc-dependent HDACs.**

|  |  | **Active sites** |
| --- | --- | --- |
| Class I | **HDAC1** | His140, His141, Gly149, Phe150, Asp174, Asp176, His178, Phe205 |
|  | **HDAC2** | His145, His146, Gly154, Phe155, Asp179, Asp181, His183, Phe210 |
| Class IIa | **HDAC5** | His832, His833, Gly841, Phe842, Asp870, His872, Asp964, Gly1004 |
|  | **HDAC9** | His782, His783, Gly791, Phe792, Asp820, His822, Asp914, Gly954 |
| Class IIb | **HDAC6** | His610, Asp649, Asp742, Tyr782 |

**Table S2. List of primers for qRT-PCR.**

|  | **Genes** | **Forward primer (5’ to 3’)** | **Reverse primer (5’ to 3’)** |
| --- | --- | --- | --- |
| Human | ***β-actin*** | GTGGCCGAGGACTTTGATTG | AGTGGGGTGGCTTTTAGGATG |
|  | ***LC3B*** | ACCATGCCGTCGGAGAAG | ATCGTTCTATTATCACCGGGATT |
|  | ***p62*** | ATCGGAGGATCCGAGTGT | TGGCTGTGAGCTGCTCTT |
|  | ***LAMP2*** | TGGCTCCGTTTTCAGCATTG | CGCTATGGGCACAAGGAAGT |
|  | ***CTSB*** | AAAAGCAGAAAACAGCTCCGC | ATCTTGCGCAGAAAGTTGGC |
|  | ***CTSD*** | GCAAACTGCTGGACATCGCTTG | GCCATAGTGGATGTCAAACGAGG |
| Mouse | ***GAPDH*** | TGTGTCCGTCGTGGATCTGA | CCTGCTTCACCACCTTCTTGAT |
|  | ***LC3B*** | GACCGGCCTTTCAAGCAG | TGGGACCAGAAACTTGGTCT |
|  | ***p62*** | TGGGCAAGGAGGAGGCGACC | CCTCATCGCGGTAGTGCGCC |
|  | ***LAMP2*** | GAGCAGGTGCTTTCTGTGTCT | ACACCCACTGCAACAGGAATA |
|  | ***CTSB*** | AAAAAGGCCTGGTTTCAGGT | GGGAGTAGCCAGCTTCACAG |
|  | ***CTSD*** | TCAGGAAGCCTCTCTGGGTA | CCCAAGATGCCATCAAACTT |
